# Relation Between Executive Function Test Performance and Treatment Outcomes During Brief Psychotherapies for Later-Life Depression

**DOI:** 10.1016/j.osep.2025.04.002

**Published:** 2025-06-11

**Authors:** Matthew S. Schurr, Yiqun T. Chen, Patrick J. Raue, Patricia A. Areán, George S. Alexopoulos, Brenna N. Renn

**Affiliations:** Department of Psychology (MSS, BNR), University of Nevada, Las Vegas, NV; Department of Psychiatry (MSS), McLean Hospital, Belmont, MA; Data Science Institute and Department of Biomedical Data Science (YTC), Stanford University, Stanford, CA; Department of Psychiatry and Behavioral Sciences (PJR, PAA), University of Washington, Seattle, WA; and the Weill Cornell Institute of Geriatric Psychiatry (GSA), Weill Cornell Medicine, White Plains, NY.

**Keywords:** Cognitive function, older adults, psychotherapy, treatment response

## Abstract

**Objectives:**

Executive dysfunction is common in later-life depression (LLD). This study examined whether: 1) executive function predicts change in depressive symptoms during brief psychotherapy and 2) performance on cognitive tests changes during psychotherapy.

**Design:**

Post hoc analysis of a noninferiority randomized clinical trial comparing 9 weekly sessions of problem-solving therapy (PST) and Engage, a streamlined psychotherapy for depression.

**Setting:**

Two-site trial at academic medical centers in Seattle, WA and New York, NY.

**Participants:**

*Participants were 150 older adults (68% women) with major depressive disorder, Mini-Mental State Examination (MMSE)* ≥*24, and cognitive test data available for primary analysis. Participants ranged in age from 60 to 89 years (*M = *70.4,* SD = *7.4).*

**Measurements:**

Cognitive measures included the Iowa Gambling Task (IGT), Wisconsin Card Sorting Test (WCST), Stroop test, Digit Span, and Hopkins Verbal Learning Test (HVLT-R). Treatment outcomes consisted of longitudinal assessment using the Hamilton Depression Rating Scale (HAM-D) and World Health Organization Disability Assessment Schedule 2.0 (WHODAS).

**Results:**

*Linear mixed effects models showed that baseline executive functioning did not predict improvement in HAM-D scores. Better Stroop performance was associated with improved WHODAS scores. Paired* t*-tests revealed pre- and post-treatment improvement in executive functioning test performance as measured by the IGT (Total Money subscale; p = 0.002) and Stroop test (p = 0.002) across 9 weeks of both treatments.*

**Conclusions:**

Baseline cognitive functions, including executive function, did not influence reduction in depressive symptoms during brief psychotherapies. Brief psychotherapies may improve aspects of executive function such as decision-making related to reward.

**ClinicalTrials.gov Identifier:**

NCT0208G201.

## INTRODUCTION

Executive dysfunction is a common feature of later-life depression (LLD) that affects more than one-third of older adults with depression^1^ and is associated with disability.^2^ Older adults with LLD often experience impairment across a variety of executive functions and related domains, including working memory, cognitive flexibility, reward response, and planning.^3,4^ Executive dysfunction is associated with poor response to antidepressant medication,^5,6^ yet it is unclear to what extent cognitive performance influences psychotherapy outcome among older adults.^7,8^ The few existing studies are mixed. For example, in two different trials of cognitive behavioral therapy (CBT) with older adults, one found poor depression outcomes^9^ and another found improved depression outcomes^10^ associated with impaired test performance on the Wisconsin Card Sorting Test (WCST).

Problem-solving therapy (PST) explicitly teaches concrete problem-solving skills and leverages behavioral activation to target common clinical presentations of LLD.^11^ PST has demonstrated efficacy in improving LLD symptoms^12^ and maintaining therapeutic gains after six months.^13^ Importantly, PST improved both symptoms of depression^14^ and disability^15^ in older adults with comorbid executive dysfunction and major depression in the Collaborative Psychotherapy study for Executive Dysfunction and Depression (COPED). Two published secondary analyses^16,17^ of this trial explored associations between cognitive test performance and psychotherapy outcomes after 12 weeks of PST or supportive therapy among adults 60 and older with depression and executive dysfunction. WCST performance improved over the course of treatment and was associated with reductions in depressive symptom severity; results did not differ across psychotherapy groups.^16^ Poorer (slower) performance on Trail Making Test, Part B at baseline was associated with higher likelihood of depression treatment response after 12 weeks of PST or supportive therapy.^17^ Taken together, these analyses of the COPED trial suggest that patients with executive impairment may reduce their depressive symptoms regardless of therapy type and focus, benefitting from a psychotherapy that does not rely heavily on executive functions (i.e., supportive therapy) as well as a highly structured psychotherapy such as PST which is designed to support executive abilities.^17^ Between the small number of studies and the inconsistent pattern of findings, however, the relation between cognitive functioning and psychotherapy for LLD remains an open question.

Engage is a newer streamlined behavioral intervention for LLD grounded in neurobiological constructs. An assumption central to Engage is that dysfunction of the positive valence systems disrupts reward-related processes and leads to abandonment of rewarding activities and ultimately to clinical symptoms of LLD.^18,19^ Engage has been shown to be noninferior to PST at improving depressive symptoms in older adults with LLD.^20^ Engage increases rewarding or meaningful stimuli (e.g., social activities, physical exercise) and simultaneously addresses behavioral barriers of negativity bias (selective attention to negative information and experience), apathy (lack of arousal and motivation), and inadequate emotional regulation.^21^ Both PST and Engage are structured interventions that seek to bypass or ameliorate limitations posed by the executive dysfunction that often accompanies LLD. However, reward approach and activation, prominent treatment mechanisms in both PST and Engage, still inherently require executive skills (e.g., cognitive flexibility, problem solving, inhibition of irrelevant stimuli). Given the prominent role executive dysfunction plays in LLD presentations, it is important to understand how executive functioning relates to clinical outcomes during behaviorally-based treatment approaches such as PST and Engage to better guide treatment selection and optimize outcomes.

## OBJECTIVES

The goals of this study are two-fold: 1) to identify tests of executive functioning and other cognitive abilities that may predict change in clinical outcomes of depressive symptoms and disability over a course of PST or Engage treatment, and; 2) identify changes in executive and other cognitive test performance that occur over the course of psychotherapy.

## METHODS

### Study Overview

This post hoc analysis examined previously collected data from a two-center, noninferiority randomized clinical trial comparing Engage and PST (NCT NCT02086201). Design, procedures, and primary outcomes of the parent study are detailed elsewhere.^20^ Participants received nine weekly sessions of either Engage or PST delivered by trained community mental health therapists between 2014 and 2019.

### Participants

Individuals were eligible for the parent trial if they were: 1) older adults (≥60 years old); 2) experiencing an active unipolar, nonpsychotic major depressive episode (assessed via SCID^22^ and eligible if Montgomery Asberg Depression Rating Scale [MADRS]^23^ ≥ 20); 3) not likely to have dementia (Mini-Mental State Examination (MMSE)^24^ ≥24; see Table 1); and 4) either not taking antidepressant medication, or on a stable dose of an antidepressant for 12 weeks and with no plans to change. Exclusion criteria were: 1) active suicidal ideation or plan; 2) psychiatric diagnoses other than unipolar, nonpsychotic major depressive disorder or generalized anxiety disorder; 3) concurrent individual psychotherapy or psychopharmacological treatment other than a stable course of antidepressants and/or mild doses of benzodiazepines (note: cholinesterase inhibitors were an outright exclusion).

### Measures

#### Demographics

Demographic information included age at the time of treatment start, gender, race, ethnicity, and years of education.

#### Outcome measures

The primary outcome was the Hamilton Depression Rating Scale (HAM-D),^25^ a 24-item, clinician-rated scale of depressive symptom severity. Each item assesses the presence and severity of a depressive symptom (e.g., depressed mood, suicide, agitation) on 0–2, 0–3, and 0–4 rating scales. Lower scores represent an absence or lower severity of the measured symptom. In addition to using raw HAM-D summary scores, we also considered two alternative categorized versions of HAM-D scores: treatment response, defined as a reduction of more than 50% from the baseline HAM-D score at week 9; and HAM-D remission, defined as achieving a HAM-D score of ≤10 at week 9. A secondary outcome of subjectively-reported disability was measured using a 12-item version of the World Health Organization Disability Assessment Schedule 2.0 (WHODAS 2.0).^26^ The WHODAS assesses six domains of disability (understanding and communicating; getting around; self-care; getting along with other; household and work activities; and participation in society) on a 5-point Likert scale (1 = *none* to 5 = *extreme or cannot do*). Using a simple sum scoring method, the WHODAS yields a possible score range of 12–60, with higher scores representing more disability. Internal consistency of baseline WHODAS scores in the sample was good, Cronbach’s alpha = .81. Both the HAM-D and WHODAS were administered at baseline and at weeks 2, 4, 6, 8, and 9.

#### Cognitive measures

Cognitive functions were assessed at baseline (week 0) and post-treatment (week 9). The Iowa Gambling Task, version 2 (IGT),^27^ a test of reward-based decision making, was administered on a computer. Total money earned was used as our outcome, with higher positive or less negative values reflecting less money “lost” and therefore suggesting more conservative or advantageous choices over the 100 trials. The Wisconsin Card Sorting Test, computer version 4, research edition (WCST)^28^ measured cognitive flexibility. Total errors were used as a marker of cognitive inflexibility (i.e., greater number of errors reflective of greater perseveration and poorer behavior self-monitoring). This computerized version is modeled off the 128-card version of the WCST but no definitive equivalence data are available for the computerized versus standard versions.^28,29^ Two other executive function tests were administered by a trained research assistant using standard paper-and-pencil administration. The Stroop Color and Word Test (Stroop)^30^ is a test of response inhibition. The color-word score was used as a measure of the ability to inhibit cognitive interference; higher scores are reflective of better performance (i.e., number of items properly named within the allotted time frame). Digit Span-backward (DSB) tested working memory and auditory attention. Finally, the Hopkins Verbal Learning Test-Revised (HVLT-R)^31^ assessed verbal memory and retention. Alternate forms were administered at baseline and week 9 to reduce practice effects. We used two scores from the HVLT-R: delayed recall (total number of words freely recalled) and a retention proportion calculated as the ratio of total number of words freely recalled during the delayed recall trial to the number of words freely recalled during the final trial of immediate recall.

### Data Analyses

The parent study consisted of 249 patient participants; of these, 150 (60.2%) had complete baseline data for HAM-D, WHODAS, IGT, WCST, Stroop, Digit Span-backward, and HVLT (see Supplementary Figure 1 for participant flow and inclusion/exclusion in this post hoc analysis). We compared individuals with and without missing cognitive testing on demographic and descriptive variables using two-sample *t*-tests and chi-square tests depending on the variable type. All values for the therapy outcomes (HAM-D and WHODAS) and cognitive tests are reported as raw scores unless otherwise specified.

For our first research question, we used linear mixed effects models to test if baseline measures of executive and other cognitive functions would predict depressive symptomatology and disability across the 9-week treatment protocol. Total money earned (IGT), total errors (WCST), number of items correctly named during color-word interference task (Stroop), Digit Span-backward, and HVLT-R delayed recall and retention scores were used as predictors of HAM-D and WHODAS scores. Fixed effects were study week (0−9), treatment group assignment (Engage or PST), baseline cognitive measures, and demographic variables (age, gender, race, and education). Models included random intercepts for each participant and a random slope for week. Supplemental exploratory analyses similarly investigated this relationship between baseline cognitive variables and the HAM-D using logistic regression to examine categorical variables of treatment response and remission.

Paired *t*-tests accounting for participant heterogeneity assessed changes in cognitive test performance from pre- (baseline) to post-treatment (week 9). To further investigate the predictive relationship between cognitive measures at baseline and post-treatment, we employed linear mixed effects models with fixed effects for baseline cognitive measure, treatment group assignment, and changes in either HAM-D or WHODAS scores with random intercepts for each patient. Finally, supplemental exploratory analyses examined correlations between changes in cognitive measures with changes in depressive symptoms (HAM-D) and disability (WHODAS). Alpha level was set at .05 and analyses were conducted in R with stats and lme4 packages.^32,33^

## RESULTS

### Sample Characteristics

Our final analytical sample consisted of 150 patients. We summarize participants’ demographic data, outcome measures, and performance on cognitive tests in Table 1. Participants were between 60 and 89 years (*M* = 70.4, *SD* = 7.4) at the start of treatment and the majority were women (n = 102, 68%), non-Hispanic/Latino (n = 139, 92.7%), and White (n = 130, 86.7%). Baseline HAM-D scores in the sample reflected depression of moderate severity. Baseline WHODAS scores reflected mild-to-moderate difficulty with daily activities. Participants removed from data analyses were not demographically different than those who were included in the data analyses, nor were they different with regards to their HAM-D or WHODAS scores at baseline or post-treatment (see Supplementary Table 1).

### Cognitive Functioning and Treatment Outcomes

Tables 2 and 3 report the coefficients from linear mixed effects models to assess whether baseline measures of cognitive functioning (IGT, WCST, Stroop, Digit Span, and HVLT-R) predicted treatment outcomes (Table 2 for HAM-D scores; Table 3 for WHODAS scores) across the 9 weeks of treatment. Both outcomes improved over time, with an average decrease of 1.14 (1.03, 1.26) units per week for HAM-D scores and an average decrease of 0.53 (0.42, 0.65) units per week for WHODAS scores. After controlling for duration of the treatment and individual heterogeneity, only performance on the HVLT-R Delayed Recall task was associated with change on the HAM-D, such that better HVLT performance (more words freely recalled) predicted improvement (less symptom intensity) on the HAM-D. Similarly, HVLT-R Delayed Recall task was also associated with improvement on the WHODAS. Stroop test performance was also associated with improvement on the WHODAS. None of the other baseline cognitive measures predicted WHODAS scores. No cognitive tests predicted categorical depression response or remission as assessed by changes in the HAM-D (Supplementary Table 2).

### Differences in Cognitive Test Performance Across Treatment

We tested for a difference in means between pre- (baseline) and post-treatment (week 9) across all cognitive measures using a paired *t*-test accounting for participant heterogeneity. There were statistically significant improvements in performance on IGT (total money subscale; *t* = −2.88, p = 0.002, Cohen’s d = 0.26) and the Stroop test (color-word interference task; *t* = −3.20, p = 0.002, Cohen’s d = 0.23) between baseline and week 9 (see Table 1). These improvements remained statistically significant after accounting for changes in depressive symptoms (HAM-D scores) and subjective disability (WHO-DAS); see details in Table 4. There was no indication that change in cognitive measures across pre- and post-treatment were associated with either changes in HAM-D or WHODAS scores (Supplementary Table 3).

## CONCLUSIONS

The principal finding of this study is that most tests of baseline cognitive test performance, including performance on tests of executive functioning, did not influence treatment outcomes of depression or disability during two brief psychotherapies. Individuals receiving Engage or PST evidenced improvements in the intended clinical outcomes regardless of cognitive test performance at the beginning of treatment. The second aim of the study was to assess changes in performance on executive functioning measures that occurred across the 9-week treatments. Reward-based decision making (assessed using the IGT) and response inhibition and response selection (assessed using the Stroop) both improved over the course of psychotherapy. These improvements remained significant after controlling for change in depressive symptoms.

The findings from our first research question suggest that a greater number of words recalled during HVLT-R delayed recall was associated with lower depressive symptoms and disability ratings at post-treatment. However, HLVT retention did not significantly predict either outcome. Thus, it may be that a raw memory retrieval ability contributes in some way to psychotherapy outcomes, but should be contextualized relative to learning (encoding). Other tests of baseline cognitive performance did not influence clinical outcome of depression during Engage or PST. Stroop performance was associated with improvement in disability on the WHODAS in our sample. These findings are best contextualized relative to other studies examining performance on select tests of executive functioning−specifically those measured by set shifting−and relation to psychotherapy treatment outcomes.^10,17^ Specifically, some studies among older adults with depression found that baseline impairment in select cognitive tests (Trail Making Test Part B^17^, WCST^10^) predicted greater depression symptom improvement.^10,17^ However, this work is not consistently replicated; primary analyses of the COPED study failed to find any moderating effect of baseline performance on the WCST, Stroop, or a few other cognitive tests on HAM-D scores after PST or supportive therapy.^14^ These studies encompass a range of psychotherapies (PST, supportive therapy, and CBT) which vary in their focus on skill-building and accommodating difficulties introduced by the presence of executive dysfunction. One area of difference is these prior studies specifically included participants with executive dysfunction^17^ or mild cognitive impairment (MCI),^34^ which the current study did not explicitly include. Cognition in LLD is generally understudied,^8^ and the vast majority of studies focus on younger adults.^35^ Given that older adults with LLD often present with executive dysfunction,^3,4^ which contributes to disability and impedes beneficial outcomes from both psychotherapy and pharmacological treatments,^5,36^ PST and Engage may offer appropriate treatment options for these individuals. Future work to understand the heterogeneity of LLD presentations—particularly among those with cognitive impairment—and psychotherapy outcomes is warranted.

Improved aspects of executive function test performance (reward-based decision making and response inhibition and selection) over the course of psychotherapy aligns with an emerging body of research showing similar changes in the Trail Making Test B^37^ and the Stroop test.^16^ Here too, findings are inconsistent across studies,^37^ which suggests that different aspects of executive functioning (e.g., cognitive flexibility, response inhibition, reward response) may be differentially influenced by LLD or by psychotherapy, and thus be optimally served by tailored treatment approaches that target the suspected behavioral sequelae associated with cognitive deficits. For example, we interpreted net improvement in total money earned on the IGT as an improvement in reward-based decision making after 9 weeks of psychotherapy. There may be more nuance required of this, however, as another study of older adults linked apathy in depression to a more rationale and conservative response style on the IGT−akin to reduced reward sensitivity−compared to nonapathetic older adults with depression, who made riskier selections on the IGT.^38^ This past work examined IGT during a antidepressant medication (escitalopram) trial and found deteriorating performance on the IGT across time; we found the opposite pattern of improved performance over 9 weeks of psychotherapy. We took care to adjust for mood symptoms in order to better isolate treatment-related improvement in cognition; that is, we wanted to ascertain if the changes in cognitive test performance were associated with treatment and not simply an epiphenomena of reduced depression or disability severity.^39^ Our findings of change in cognitive test performance may reflect a specific effect of psychotherapy on a focal aspect of executive function; it may also reflect type I error in an exploratory study using multiple comparison. Notably, tasks such as the WCST and IGT rely heavily on figuring out a strategy; learning and remembering the principle of success render repeated measurement difficult to interpret. However, we also found improvement on the Stroop test, which by some reports is not susceptible to practice effects.^40^

Taken together, the etiology of both cognitive decline in LLD^41^ and change in cognition associated with depression treatment remains unclear. Some of this confusion is due to a small body of research with methodological heterogeneity and limited focus on older adults with LLD, particularly undergoing psychotherapy.^8,35,37,42,43^ As the purpose of the parent study was not to test psychotherapy effects on cognitive outcomes, the present analyses are exploratory and include limitations. First, the parent study demonstrated an overall pattern of depressive symptom improvement with noninferiority between Engage and PST, so we had reduced variance in treatment outcomes to interrogate associations between cognitive functioning and depressive symptoms. The parent study used a score of MMSE ≥ 24 for inclusion, meaning many participants were likely within normal limits of cognitive functioning. The MMSE is not sensitive to detecting MCI or executive dysfunction^44,45^ and our sample may include individuals with MCI,^34^ but the average level of executive functioning among indivdiuals in our study is not likely to be impaired enough to pose a significant barrier to psychotherapy outcomes. Similarly, changes in cognitive measures from baseline to week 9, though statistically significant, may not represent a clinically meaningful change in cognitive functioning. This may also reflect the cognitively intact sample, where average cognitive test performance across the sample was within an average range or higher, and performance on the WCST near ceiling level. Other relevant cognitive functions, such as verbal fluency, were not included in the present analysis. Given the exploratory nature of these analyses, findings may simply be due to chance in the context of multiple comparisons. The sample was majority non-Hispanic and White and more highly educated than the majority of US adults, which limits generalizability of these findings. Next steps in this work should seek to enrich their samples with individuals with executive dysfunction (such as was done in the COPED trial)^15^ and prespecify a cognitive test or composite as the primary outcome.^39^ Additionally, there is emerging work that different patterns of nonlinear depressive symptom change over psychotherapy are associated with specific cognitive outcomes; more work to understand the complexity of these associations is warranted.^46^ Future research may also consider whether psychotherapy outcomes vary by diagnostic categories, such as among adults with MCI. One approach may be to further investigate psychotherapy outcomes among individuals with sustained cognitive impairment compared to those with improvement or intact/normal cognitive functioning. This would better establish whether the proposed benefits of these brief treatment protocols for LLD are maintained in these populations and better assess any improvements in cognitive performance on neuropsychological measures across psychotherapy. Finally, this post hoc analysis did not account for other potential moderators of the relation between cognition and treatment outcomes, including number of prior depressive episodes, co-occurring health conditions, or medication use (although trial inclusion required either no antidepressant medication or stability in dosage).

Overall, our results are consistent with the reward-based conceptualization of depression. Disruption to reward anticipation and processing, along with other positive valence systems (i.e., decision making, and action and effort selection), is a central process in the maintenance of LLD.^21^ Engage was specifically designed to address these disruptions by implementing “reward exposure”−that is, structured engagement in pleasurable or meaningful activities−as the primary treatment mechanism to recondition the reward system and improve LLD.^21^ PST teaches structured problem-solving skills and strengthens planning and follow-through, enabling patients to be exposed to rewarding activities.^15^ Thus, both treatments may result in the same functional outcome through slightly different approaches in session. It appears that highly structured, brief treatment protocols such as Engage and PST work as intended to improve depressive symptoms among older adults. While antidepressant medication remains the most common treatment for LLD in real world practice,^47^ this is not aligned with older adult patient treatment preferences^48,47^ and is likely to yield poor response to treatment in those with comorbid executive dysfunction.^5,6^ An open question is whether improvement in cognitive functioning is a function of general improvement in depressive symptoms, specific to a compensatory framework provided by particular psychotherapies, or merely practice effects of test repetition. Future work is needed to understand whether LLD patients presenting with specific neurocognitive deficits respond differentially to different therapeutic approaches (e.g., PST to strengthen a positive problem-solving orientation; Engage to strengthen planning related to reward response). Such research can further define LLD presentations and guide clinical decision making among geriatric mental health providers.

## Supplementary Material

1

SUPPLEMENTARY MATERIALS

Supplementary material associated with this article can be found in the online version at doi:10.1016/j.osep.2025.04.002.

## Figures and Tables

**TABLE 1.** Demographic Characteristics and Assessment Measures at Baseline and Week 9


Sample Characteristics (N = 150)	*M (SD)* or *n (%)*	p-*Values*

Age (years), *M* (*SD*)	70.4 (7.4)	
Median (Range)	69 (60–89)	
Gender,^a^ *n* (*%*)	102 (68.0%)	
Women	47 (31.3%)	
Men		
Race,^b^ *n* (*%*)	130 (86.7%)	
White	8 (5.3%)	
African American/Black	3 (2.0%)	
Asian American	7 (4.7%)	
Other not listed above		
Ethnicity,^c^ *n* (*%*)	8 (5.3%)	
Hispanic/Latino	139 (92.7%)	
Non-Hispanic/Latino		
Education (years)		
*M* (*SD*)	16.1 (2.5)	
Range	12–25	
MMSE		
*M* (*SD*)	28.7 (1.2)	
Range	25–30	
HAM-D, *M* (*SD*)		
Baseline	23.2 (4.1), range 16–37	
Week 9	11.8 (6.7), range 0–28	<0.001
WHODAS, *M* (*SD*)		
Baseline	28.0 (7.7)	
Week 9	23.4 (8.0)	<0.001
IGT,^d^ *M* (*SD*)		
Baseline	−882 (1,390)	
Week 9	−452 (1,370)	0.002
WCST,^d^ *M* (*SD*)		
Baseline	18.8 (9.0)	
Week 9	18.41 (9.65)	0.58
Stroop test,^d^ *M* (*SD*)		
Baseline	33.1 (8.9)	
Week 9	35.10 (9.31)	0.002
DSB,^d^ *M* (*SD*)		
Baseline	6.6 (2.3)	
Week 9	6.8 (2.3)	0.09
HVLT-R immediate recall,^d^ *M* (*SD*)		
Baseline	23.2 (5.2)	
Week 9	23.7 (5.5)	0.86
HVLT-R,^d^ delayed recall, *M* (*SD*)		
Baseline	8.1 (2.6)	
Week 9	8.0 (2.7)	0.23
HVLT-R retention,^d^ *M* (*SD*)		
Baseline	34.6% (8.0%)	
Week 9	35.0% (8.7%)	0.90

a One participant (0.7%) had no gender information recorded.

b Two participants had no racial data recorded.

c Three participants had no ethnicity data recorded.

d Paired t-tests with unequal variance are used to compare the observed cognitive performance for participants with observations at baseline and Week 9.

Abbreviations: MMSE = mini-mental state examination; HAM-D = Hamilton depression rating scale; WHODAS 2.0 = world health organization disability assessment schedule 2.0; IGT = Iowa gambling task - total money earned; WCST = Wisconsin card sorting test - total errors; Stroop test = color-word score; DSB = digit span-backward; HVLT-R immediate recall = Hopkins verbal learning test (revised) - immediate recall, total words learned over three trials; HVLT-R delayed recall = HVLT-R delayed recall total score; HVLT-R Retention = proportion of words retained, expressed as a percentage, of HVLT-R delayed recall to immediate recall scores.

**TABLE 2.** Associations Between Baseline Cognitive Measures and HAM-D Scores Over the Course of Treatment


Predictor	*β*_1_	p-Values	95% CI	*β*_*week*_	p-Values	95% CI

IGT	−0.0002	0.46	[−0.0006, 0.0003]	−1.11	<0.001	[−1.22, h1.00]
WCST	0.03	0.36	[−0.04, 0.10]	−1.11	<0.001	[−1.22, −1.01]
Stroop	−0.02	0.57	[−0.08, 0.05]	−1.09	<0.001	[−1.19, −0.99]
DSB	−0.094	0.42	[−0.35, 0.16]	−1.10	<0.001	[−1.20, −1.00]
HVLT-R delayed recall	−0.24	0.03	[−0.48, −0.01]	−1.10	<0.001	[−1.20, −1.00]
HVLT-R retention	−3.26	0.34	[−10.0, 3.49]	−1.10	<0.001	[−1.20, −1.00]

*Note:* Predictors refer to the baseline values of the following cognitive measures: IGT = Iowa Gambling Task - total money earned; WCST = Wisconsin Card Sorting Test - total errors; Stroop = color-word score; DSB = digit span-backward; HVLT-R delayed recall = Hopkins Verbal Learning Test (revised) - delayed recall total score; HVLT-R Retention = proportion of word retained, expressed as a percentage, of HVLT-R delayed recall to immediate recall scores. The outcome of interest is HAM-D (Hamilton Depression Rating Scale) scores. Coefficients are estimated from a linear mixed model with treatment group, demographic variables (age, gender, race, education), cognitive score (*β*_1_) and week (*β*_week_) as fixed-effects predictors, random intercepts for each participant, and a random slope for the week predictor. Treatment consisted of up to nine weekly sessions of a brief psychotherapy.

**TABLE 3.** Associations Between Baseline Cognitive Measures and WHODAS Scores Over the Course of Treatment


Predictor	*β*_1_	p-Values	95% CI	*β*_*week*_	p-Values	95% CI

IGT	0.0005	0.23	[−0.0003, 0.001]	−0.51	<0.001	[−0.63, −0.40]
WCST	0.07	0.28	[−0.05, 0.19]	−0.52	<0.001	[−0.62, −0.42]
Stroop	−0.17	0.002	[−0.28, −0.06]	−0.48	<0.001	[−0.58, −0.38]
DSB	−0.34	0.11	[−0.77, 0.08]	−0.48	<0.001	[−0.58, −0.39]
HVLT-R delayed recall	−0.52	0.01	[−0.92, −0.13]	−0.48	<0.001	[−0.58, −0.38]
HVLT-R retention	−7.02	0.24	[−18.9, 4.84]	−0.48	<0.001	[−0.58, −0.38]

*Note:* Predictors refer to the baseline values of the following cognitive measures: IGT = Iowa Gambling Task - total money earned; WCST = Wisconsin Card Sorting Test - total errors; Stroop = color-word score; DSB = digit span-backward; HVLT-R delayed recall = Hopkins Verbal Learning Test (revised) - delayed recall total score; HVLT-R retention = proportion of word retained, expressed as a percentage, of HVLT-R delayed recall to immediate recall scores. The outcome of interest is WHODAS 2.0 (World Health Organization Disability Assessment Schedule 2.0) scores. Coefficients are estimated from a linear mixed model with treatment group, demographic variables (age, gender, race, education), cognitive score (*β*_1_)and week (*β*_week_) as fixed-effects predictors, random intercepts for each participant, and a random slope for the week predictor. Treatment consisted of up to nine weekly sessions of a brief psychotherapy.

**TABLE 4.** Estimated Changes in Cognitive Measures From Baseline to Week 9 (End of Treatment)


	Controlling for Changes in HAM-D			Controlling for Changes in WHODAS		
Cognitive Measures	Estimated Changes	p-Values	95% CI	Estimated Changes	p-Values	95% CI

IGT	497	0.001	[198, 796]	498	0.001	[198, 797]
WCST	−0.57	0.5	[−2.31, 1.16]	−0.48	0.6	[−2.19, 1.24]
Stroop	1.76	0.001	[0.70, 2.83]	1.67	0.002	[0.60, 2.74]
DSB	0.30	0.06	[−0.01, 0.60]	0.30	0.06	[−0.01, 0.60]
HVLT-R delayed recall	−0.24	0.24	[−0.63, 0.16]	−0.27	0.18	[−0.67, 0.13]
HVLT-R retention	0.001	0.50	[−0.002, 0.003]	0.001	0.55	[−0.002, 0.003]

Abbreviations for measures: IGT = Iowa Gambling Task - total money earned; WCST = Wisconsin Card Sorting Test - total errors; Stroop = color-word score; DSB = digit span-backward; HVLT-R delayed recall = Hopkins Verbal Learning Test (revised) - delayed recall total score; HVLT-R retention = proportion of word retained, expressed as a percentage, of HVLT-R delayed recall to immediate recall scores; HAM-D = Hamilton Depression Rating Scale; WHODAS = World Health Organization Disability Assessment Schedule 2.0. Analyses are adjusting for (i) treatment group; and (ii) changes in either HAM-D or the WHODAS scores using a linear mixed model with random intercepts for each participant.

## Data Availability

Raw data were generated as part of NCT02086201 and maintained by investigators at Weill Cornell Medicine. Derived data supporting the findings of this study are available from the corresponding author [BNR] on request.

## Highlights

### What is the primary question addressed by this study?

What is the association between cognitive test performance and depression outcomes during brief psychotherapy for late-life depression?

### What is the main finding of this study?

Baseline executive functioning did not influence improvements in depression or disability during two brief psychotherapies among older adults. Reward-based decision making and response inhibition and response selection both improved over the course of psychotherapy, even after controlling for change in depressive symptoms.

### What is the meaning of the finding?

Brief psychotherapies tailored for late-life depression may lead to significant improvements in depressive symptomatology and disability, even in the context of variable cognitive test performance.
