# Supplementary material for: Relation Between Executive Function Test Performance and Treatment Outcomes During Brief Psychotherapies for Later-Life Depression
Source: Am J Geriatr Psychiatry Open Sci Educ Pract. Author manuscript; Available in PMC 2025 Nov 26. (PMC12646658; doi:10.1016/j.osep.2025.04.002)

Supplementary Table S1

Participant Characteristics and Outcomes Stratified by Participants Excluded and Included in the Analytic Sample

**Participant Characteristics**	**Analytic Sample (*n*=150)**	**Excluded Sample (*n*=99)**	**Overall (*N*=249)**	**p-values**[a]
**Age (years),**[b] *M* (*SD*)	70.4 (7.4)	69.8 (7.3)	70.1 (7.4)	0.54
Median (Range)	69 (69 – 89)	68 (60 – 88)	69 (60 – 89)	
**Gender,**[c] *n (%)*				0.90
Women	102 (68.0%)	77 (68.0%)	179 (67.8%)	
Men	47 (31.3%)	37 (31.3%)	84 (31.8%)	
Not available	1 (0.7%)	0	1 (0.4%)	
**Race**,[c] *n (%)*				0.95
White	130 (86.7%)	102 (89.5%)	232 (87.9%)	
African American/Black	8 (5.3%)	5 (4.4%)	13 (4.9%)	
Asian American	3 (2.0%)	1 (0.9%)	4 (1.5%)	
Other not listed above	7 (4.7%)	5 (4.5%)	12 (4.5%)	
Not available	2 (1.3%)	1 (0.9%)	3 (1.1%)	
**Ethnicity**,[c] *n (%)*				0.47
Hispanic/Latino	8 (5.3%)	3 (2.6%)	11 (4.2%)	
Non-Hispanic/Latino	139 (92.7%)	110 (96.5%)	249 (94.3%)	
Not available	3 (2.0%)	1 (0.9%)	4 (1.5%)	

Participant Characteristics	Analytic Sample (*n*=150)	Excluded Sample (*n*=99)	Overall (*N*=249)	p-values[a]
**Education**[b] (years)				0.68
M (*SD*)	16.1 (2.5)	16.0 (2.8)	16.1 (2.6)	
Range	12-25	6-22	6-25	
**Treatment Group**,[c] n (%)				0.42
Engage	73 (48.7%)	56 (56.6%)	129 (51.8%)	
PST	77 (51.3%)	43 (43.4%)	120 (48.2%)	
**MMSE**,[b] *M* (*SD*)	28.7 (1.2)	28.7 (1.5)	28.7 (1.3)	0.86
**HAM-D**,[b] *M* (*SD*)				
Baseline	23.2 (4.1)	23.2 (4.5)	23.2 (4.3)	0.99
Post-treatment (week 9)	11.8 (6.7)	12.9 (8.0)	12.2 (7.2)	0.30
**WHODAS**,[b] *M* (*SD*)				
Baseline	28.0 (7.7)	23.4 (8.1)	28.4 (7.9)	0.95
Post-treatment (week 9)	23.4 (8.0)	24.5 (8.1)	23.8 (8.1)	0.33

Table Footnotes

[a] *p*-values used to assess any difference in demographic characteristics between the included and excluded samples in our study.

[b] Two-sample t-test with unequal variance.

[c] Chi-square contingency-table test.

Abbreviations: PST = problem-solving therapy; MMSE = Mini-Mental State Examination (administered at study eligibility screening); HAM-D = Hamilton Depression Rating Scale; WHODAS 2.0 = World Health Organization Disability Assessment Schedule 2.0

Supplementary Table S2

Associations Between Baseline Cognitive Measures and Categorical HAM-D Outcomes at Post-Treatment

	HAM-D Response			HAM-D Remission		
Predictor	Odds ratio	*p*-values	95% CI	Odds ratio	*p*-values	95% CI
IGT	1.00	0.94	[0.99, 1.00]	1.00	0.77	[0.99, 1.00]
WCST	0.98	0.37	[0.95, 1.02]	0.99	0.69	[0.96, 1.03]
Stroop	0.99	0.54	[0.95, 1.02]	0.98	0.32	[0.95, 1.02]
DSB	1.01	0.86	[0.88, 1.16]	1.04	0.59	[0.9, 1.2]
HVLT-R Delayed Recall	1	0.99	[0.88, 1.13]	0.99	0.87	[0.87, 1.12]
HVLT-R Retention	1.83	0.74	[0.05, 70.84]	0.82	0.91	[0.02, 32.07]

Note. IGT = Iowa Gambling Task - Total Money Earned; WCST = Wisconsin Card Sorting Test - Total Errors; DSB = Digit Span-Backward; HVLT-R Delayed Recall = Hopkins Verbal Learning Test (Revised) - Delayed Recall Total Score; HVLT-R Retention = proportion of word retained, expressed as a percentage, of HVLT-R Delayed Recall to Immediate Recall scores. Hamilton Depression Rating Scale (HAM-D) response is defined as a reduction of more than 50% from the baseline HAM-D score at week 9. HAM-D remission is defined as achieving a HAM-D score of ≤10 at week 9. The coefficients presented are estimated from a logistic regression model that includes treatment group, demographic variables (age, gender, race), and baseline cognitive scores as predictors. Pre-treatment scores are baseline scores collected immediately prior to treatment start. Post-treatment scores reflect the Week 9 assessment timepoint.

Supplementary Table S3

Correlations between Changes in Cognitive Measures and Clinical Outcomes from Pre- to Post-Treatment.

Change in cognitive measures	Correlation with change in HAM-D (*p*-value)	Correlation with change in WHODAS (*p*-value)	Partial correlation with change in WHODAS, controlling for change in HAM-D (*p*-value)
IGT	-0.04 (0.65)	-0.11 (0.22)	0.08 (0.41)
WCST	-0.09 (0.30)	0.10 (0.28)	0.07 (0.47)
Stroop	-0.11(0.20)	0.03 (0.07)	-0.08 (0.39)
DSB	0.09 (0.38)	-0.01 (0.80)	-0.11 (0.22)
HVLT-R Delayed Recall	0.06 (0.54)	0.06 (0.49)	0.07 (0.41)
HVLT-R Retention	0.06 (0.49)	0.09 (0.29)	0.07 (0.41)

Note. Abbreviations for measures: IGT = Iowa Gambling Task - Total Money Earned; WCST = Wisconsin Card Sorting Test - Total Errors; Stroop = color-word score; DSB = Digit Span-Backward; HVLT-R Delayed Recall = Hopkins Verbal Learning Test (Revised) - Delayed Recall Total Score; HVLT-R Retention = proportion of word retained, expressed as a percentage, of HVLT-R Delayed Recall to Immediate Recall scores; HAM-D = Hamilton Depression Rating Scale; WHODAS = World Health Organization Disability Assessment Schedule 2.0. Pre-treatment scores are baseline scores collected immediately prior to treatment start. Post-treatment scores reflect the Week 9 assessment timepoint.

Supplementary Figure S1

Participants from the Parent Trial[20] Included and Excluded from this Secondary Analysis

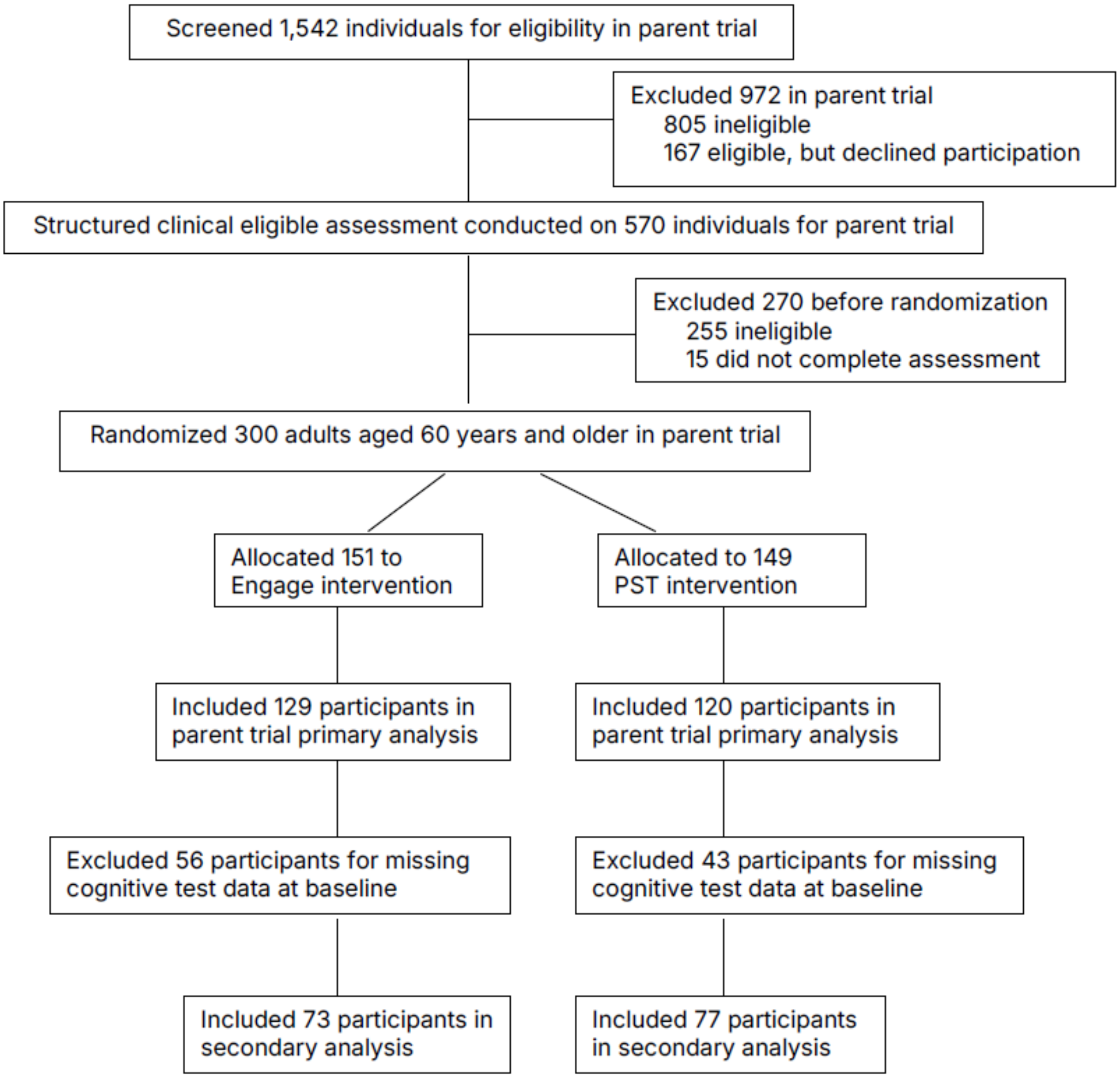

Supplement: 1 [file NIHMS2109207-supplement-1.pdf]
